# RBM17 Promotes the Chemoresistance of Oral Squamous Cancer Cells Through Checkpoint Kinase 1

**DOI:** 10.3390/ijms26073127

**Published:** 2025-03-28

**Authors:** Miyuka Nakahara, Ryosuke Arai, Isao Tokuoka, Kazuhiro Fukumura, Akila Mayeda, Masakazu Yashiro, Hirokazu Nakahara

**Affiliations:** 1 Department of Oral and Maxillofacial Surgery, Osaka City University Graduate School of Medicine, Osaka 545-8585, Japan; 2 Department of Oral and Maxillofacial Surgery, Osaka Metropolitan University Graduate School of Medicine, Osaka 545-8585, Japan; d21mb003@st.osaka-cu.ac.jp (R.A.); tokuoka.isao@omu.ac.jp (I.T.); 3 Oncology Innovation Center, Fujita Health University, Toyoake 470-1192, Japan; fukumura@fujita-hu.ac.jp (K.F.); mayeda@fujita-hu.ac.jp (A.M.); 4 xFOREST Therapuetics, Co., Ltd., Kyoto 602-0841, Japan; 5 Molecular Oncology and Therapeutics, Osaka Metropolitan University Graduate School of Medicine, Osaka 545-8585, Japan; i21496f@omu.ac.jp

**Keywords:** RBM17/SPF45, Chk1, oral squamous cell carcinoma, chemotherapy, chemoresistance

## Abstract

Oral squamous cell carcinoma (OSCC) is one of the most common types of cancer in the head and neck region. In advanced stages of OSCC, chemotherapy is commonly used for treatment, despite some cancer cells having low sensitivity to anticancer drugs. We focused on RBM17/SPF45 as an essential drug-sensitizing factor in the context of malignant cells acquiring chemoresistance. Here, we demonstrate how RBM17 affects anticancer drug resistance in OSCC and we suggest the possible mechanism underlying its effects. After exposing oral cancer cell lines to fluorouracil (5-FU) and cisplatin, but not paclitaxel, the gene and protein expression of RBM17 increased. We found that siRNA-mediated *RBM17*-knockdown of the cell lines gained a significantly higher sensitivity to 5-FU, which was remarkably followed by a decrease in the expression of checkpoint kinase 1 (CHEK1) protein, whereas treatment with a CHEK1 inhibitor did not affect RBM17 protein expression in the oral cancer cell lines. These results indicate that RBM17 is a factor involved in the development of resistance to cytotoxic chemotherapy. We propose the underlying mechanism that RBM17 promotes CHEK1 protein expression in the ATM/ATR pathway, triggering the development of chemoresistance in cancer cells.

## 1. Introduction

Oral squamous cell carcinoma (OSCC) is a common type of cancer in the oral region [1]. The Global Cancer Observatory reported that the annual incidence, mortality, and 5-year prevalence of OSCC were 389,846, 188,438, and 272,998 in 2004 (both sexes and all ages), respectively [2]. In the early stages of OSCC, surgical resection is the preferred treatment option, although it has a significant impact on the patient’s quality of life [3,4]. Radiotherapy or chemoradiotherapy is typically added as an adjuvant treatment for patients at high risk [5]. However, cancer cells may develop resistance to anticancer drugs at any stage of the disease, including at the time of diagnosis, during treatment, or upon recurrence. The development of multi-drug resistance in cancer cells during chemotherapy can be attributed to multiple mechanisms that are poorly elucidated thus far [6].

RNA-binding motif protein 17 (RBM17)/splicing factor 45 (SPF45) was first identified as a member of the spliceosome complex [7] and later found to be a regulator of alternative splicing [8]. Recently, RBM17, together with the cofactor SAP30BP, has been identified as an essential splicing factor targeting a subset of short introns [9,10], indicating that the expression of genes containing such short introns are under the control of RBM17. RBM17 is expressed at low levels in normal tissues, yet is frequently overexpressed in various cancers [11]. Several studies have demonstrated that RBM17 is responsible for chemoresistance in cancers [11,12]. The overexpression of RBM17 in cervical and ovarian cancer cells results in resistance to at least six anticancer drugs with different mechanisms. It is notable that overexpression of RBM17 does not impact the intracellular accumulation of drugs, which is well-documented as a mechanism of multidrug resistance [11,13,14].

Cytotoxic anticancer drugs exert their pharmacological effects through a variety of mechanisms, with DNA damage-induced cell death being one of the most widely recognized [15,16]. It has been observed that when lesions are formed in healthy cells, the DNA repair system, in which the DNA damage response (DDR) factors are involved, recognizes and acts upon the cells depending on the type of DNA damage. Once the cells undergo genotoxic anticancer treatment, they will undergo repair mechanisms to overcome the injury induced by these agents. However, once the damage is too severe and irreversible, apoptosis is activated. The increased DNA repair potential is linked to drug resistance and reduces chemotherapy effectiveness [17]. Checkpoint kinase 1 (CHEK1) plays a more expansive role in checkpoint activation during the DDR and normal cell cycle regulation [18]. Targeted inhibition of CHEK1 is therefore a potential strategy to alleviate multidrug resistance [19,20].

In this study, we evaluate the potential utility of the RBM17 protein as a biomarker of chemotherapy resistance. We also examine the potential of RBM17 as a therapeutic target for oral cancer.

## 2. Results

### 2.1. Inhibitory Effects of OSCC Cell Lines for 5-FU, CDDP, and PTX

**Figure 1:**
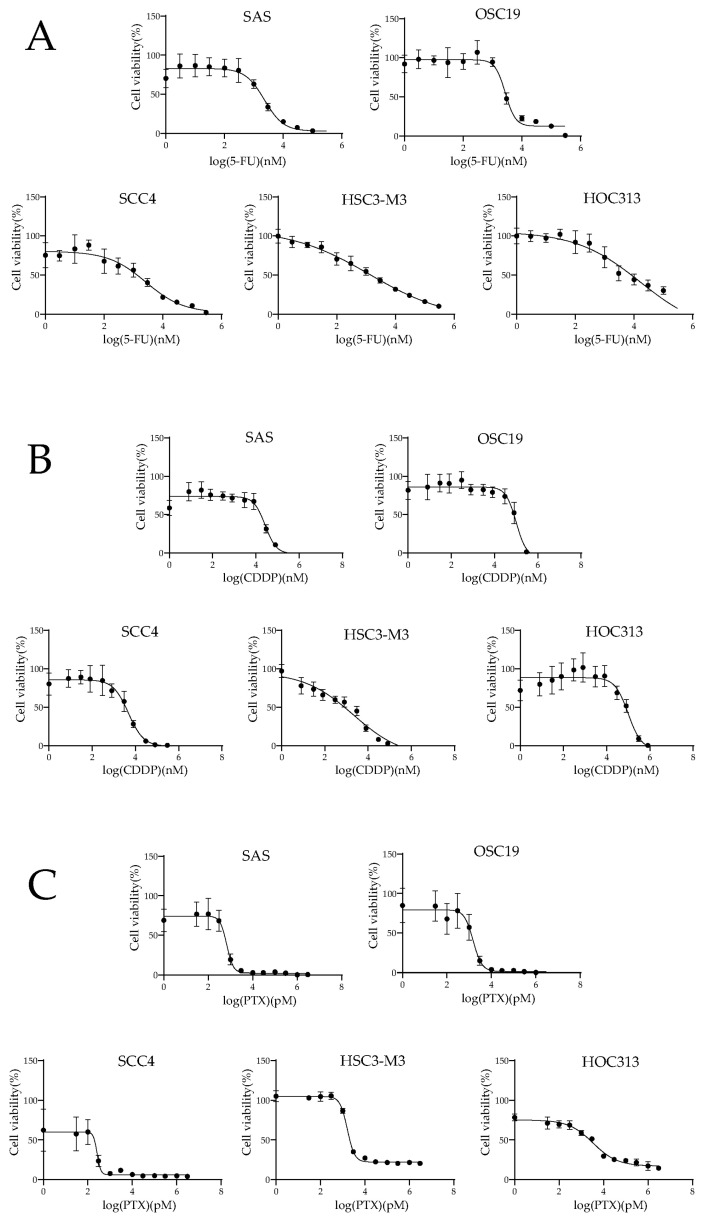
The 3-(4,5-dimethylthiazol-2-yl)-2,5-diphenyl tetrazolium bromide (MTT) assay was conducted to determine the absorbance of the indicated cell lines 48 h after the administration of anticancer drug 5-FU (**A**), CDDP (**B**), and PTX (**C**). From these data, IC50 were calculated (Table 1).

The half-maximal inhibitory concentration (IC50) was quantified via four-parameter nonlinear logistic regression. The sensitivity to fluorouracil (5-FU), cisplatin (CDDP), and paclitaxel (PTX) were investigated at concentrations of 0–300 µM, 0–800 µM, and 0–3 µM, respectively (Figure 1), and the IC50 values of 5-FU, CDDP, and PTX for each cell line were calculated (Table 1).

### 2.2. RBM17 mRNA and Protein Expressions After Cytotoxic Anticancer Drug Administration

**Figure 2:**
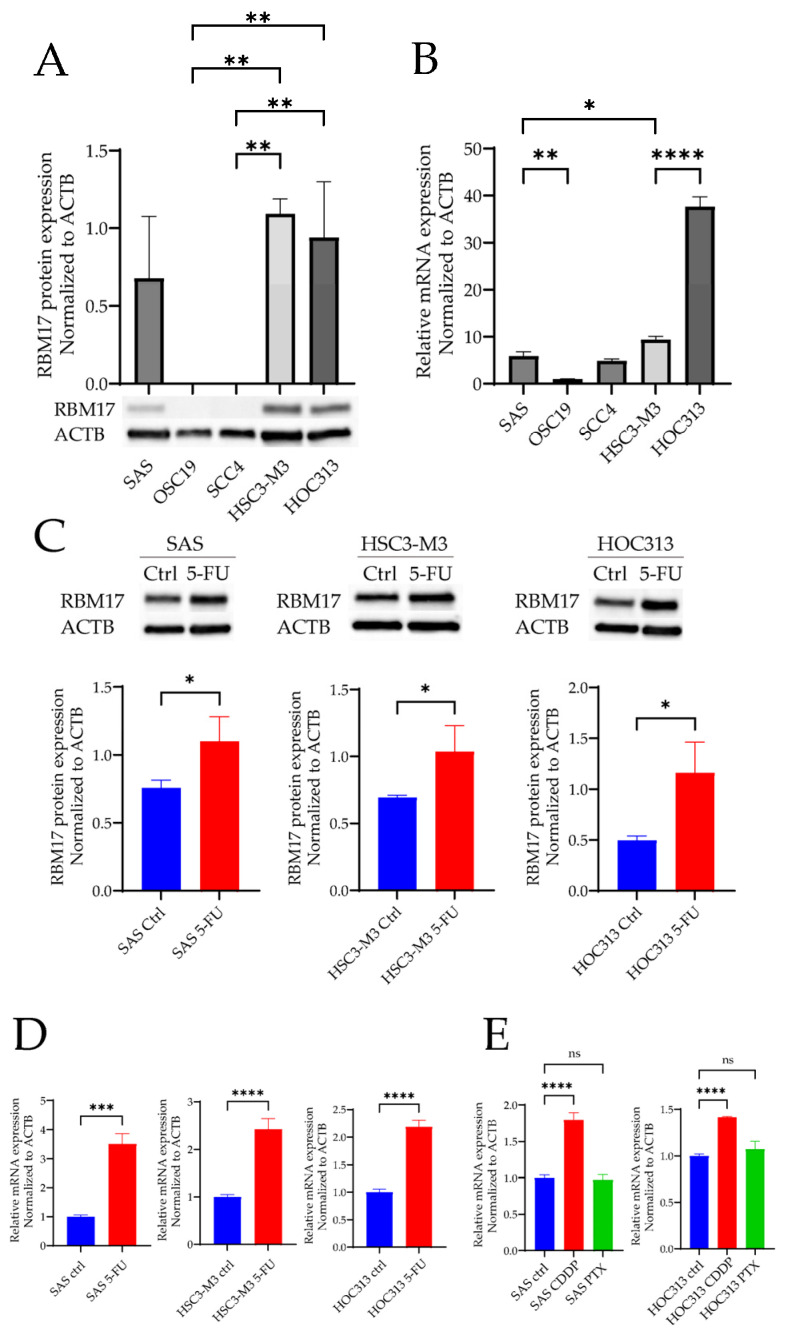
(**A**) Western blotting was performed to examine the expression of the RBM17 protein in OSCC cell lines. The intensity of the detected bands was quantified and compared using the image analysis software ImageJ 1.53k. Triplicate blots are shown in the Supplementary Materials (Figure S1A). ** *p* < 0.01 (**B**) The mRNA expression of RBM17 in OSCC cell lines was analyzed by RT-PCR. * *p* < 0.05, ** *p* < 0.01, **** *p* < 0.0001. (**C**) The 5-FU-exposure of SAS, HSC3-M3, and HOC313 cell lines for a period of seven days induced an observable increase in RBM17 protein expression. The intensity of the detected bands on Western blots was quantified and compared using the image analysis software ImageJ. Triplicate blots are shown in the Supplementary Materials (Figure S1B). * *p* < 0.05. (**D**) The 5-FU-exposure of the same cell lines for a period of seven days also induced an increase in the expression of the RBM17 mRNA. (**E**) Following a 48-h exposure to CDDP or PTX, the mRNA expression of RBM17 was found to be increased by CDDP, while no change in expression was observed by PTX in either the SAS or HSC3-M3 cell lines. *** *p* < 0.001, **** *p* < 0.0001. Each experiment had three biological replicates. All data are represented as mean ± SD.

The expression levels of RBM17 protein and RBM17 mRNA were analyzed (Figure 2A,B). RBM17 was highly expressed in HSC3-M3 and HOC313 cells. RBM17 expression was slightly higher in the SAS cells than in OSC19 and SCC4 cells.

The expression levels of RBM17 protein and RBM17 mRNA were quantified in cells following the administration of cytotoxic anticancer drugs. The expression of the RBM17 protein and mRNA were increased in the SAS, HSC3-M3, and HOC313 cell lines during the administration of 5-FU (Figure 2C,D). The RBM17 mRNA was found to be overexpressed in both SAS and HOC313 cells following the administration of CDDP. In contrast, PTX did not result in the elevation of RBM17 expression in either SAS or HOC313 cells (Figure 2E).

### 2.3. Changes in 5-FU Drug Sensitivity After RBM17 Gene Knockdown

**Figure 3:**
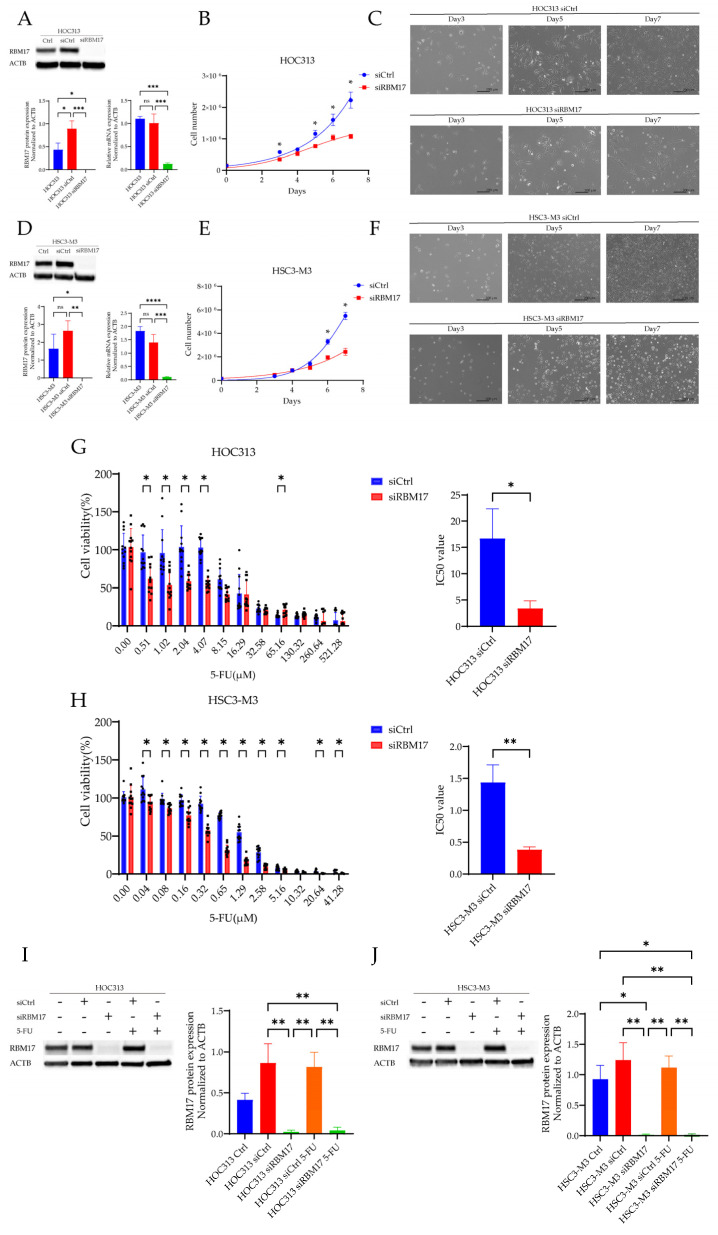
The alterations in 5-FU sensitivity upon *RBM17* gene knockdown. (**A**,**D**) Following the siRNA-mediated RBM17 knockdown in the HOC313 and HSC3-M3 cell lines, respectively, the mRNA expression was examined using RT-PCR and the protein expression was examined by Western blotting. Each experiment had three biological replicates. Triplicate blots are shown in the Supplementary Materials (Figure S2A,B). * *p* < 0.1, ** *p* < 0.01, *** *p* < 0.001, **** *p* < 0.0001. (**B**,**E**) The cytotoxic effect of *RBM17* siRNA was determined by a cell proliferation assay of the HOC313 and HSC3-M3 cells, respectively. The observed partial inhibitory effect on cell proliferation in RBM17-knockdown cells was expected since RBM17 is a general splicing factor for a subset of introns. (**C**,**F**) The status of the HOC313 and HSC3-M3 cells, respectively, were examined by microscopy three, five, and seven days following the transfection of siRNAs. A higher percentage of dead cells was observed in RBM17 siRNA treated cells compared with that in control siRNA treated cells. (**G**,**H**) The HOC313 and HSC3-M3 cell lines, respectively, were exposed to 5-FU at doses ranging from the indicated concentrations, and the resulting absorbance was quantified via an MTT assay after a seven-day incubation period. (Each black dot in the graph represents an individual trial). In addition, the IC50 of RBM17 siRNA-treated cells and the control were measured. The IC50 values for HOC313 siCtrl and HOC313 siRBM17 were 15.867 µM (12.858–19.610) and 4.054 µM (2.986–5.484), and for HSC3-M3 siCtrl and HSC3-M3 siRBM17 were 1.422µM (1.266–1.597) and 0.3806 (0.349–0.415), respectively. * *p* < 0.05, ** *p* < 0.01. (**I**,**J**) HOC313 and HSC3-M3 cells, respectively, transfected with either control siRNA or *RBM17* siRNA, were exposed to 5-FU, and the expression levels of RBM17 were determined through Western blot analysis. Each experiment had four biological replicates. Data are represented as mean ± SD. Quadruplicate blots are shown in the Supplementary Materials (Figure S2C,D). * *p* < 0.1, ** *p* < 0.01.

The RBM17 siRNA-treated cells showed a striking reduction in both the RBM17 mRNA and RBM17 protein levels in comparison to those of the control cells (Figure 3A,D). To assess the cytotoxic potential of RBM17 siRNA, a cell proliferation assay was conducted and the cell status was checked on days 3, 5, and 7 following the transfection of siRNAs (Figure 3B,C,E,F).

Following transfection with either control siRNA or *RBM17* siRNA, 5-FU was administered at a concentration approximating the IC50. In both the HSC-3-M3 and HOC-313 cell lines, the RBM17 siRNA-treated cells exhibited a significant elevation in drug sensitivity to 5-FU compared to the control cells. The drug sensitivity of the RBM17-knockdown group in the HOC313 cell line exhibited a notable change in drug sensitivity at low 5-FU concentrations, as compared to the control-knockdown cells (Figure 3G). In contrast, high drug sensitivity of the HSC3-M3 cell line was observed around the IC50 of 5-FU (Figure 3H). After RBM17 knockdown, the IC50 of 5-FU was significantly decreased in both HOC313 and HSC3-M3 (Figure 3G,H, right graph). The administration of 5-FU to RBM17-transfected HOC-313 and HSC-3-M3 cell lines did not result in an increased expression of RBM17 protein (Figure 3I,J).

### 2.4. The Relationship Between RBM17 and CHEK1 Protein Expression

**Figure 4:**
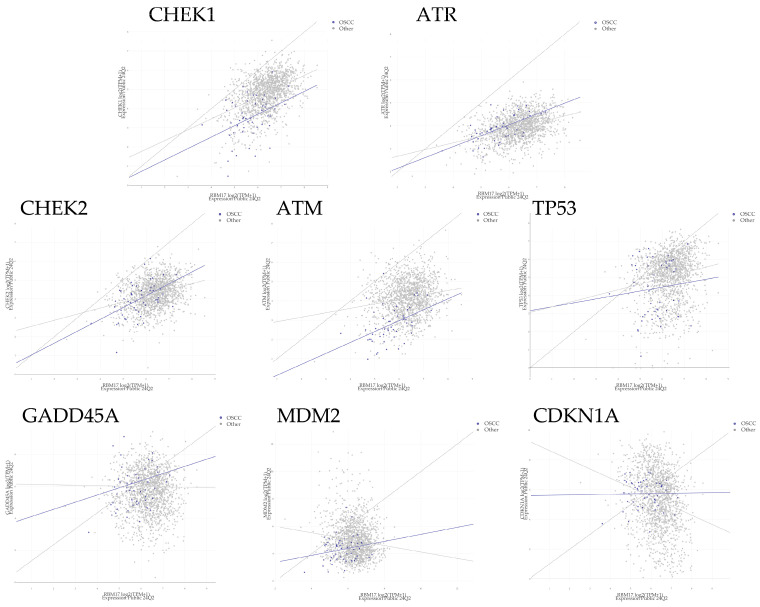
The relationship between RBM17 and DDR-related genes. Correlation analyses between RBM17 and DDR-related genes were conducted using the DepMap portal database and the results demonstrated a statistically significant correlation between the expression of RBM17 and CHEK1 genes (*p* < 0.001). The lines represent regression lines (gray lines: non-OSCC cancer cells, blue lines: OSCC cells).

The relationship between RBM17 and DDR-related gene expressions was evaluated using the Pearson’s and Spearman’s rank correlation coefficients with data from the DepMap database (Figure 4). The strongest correlations were observed between RBM17 and CHEK1, with the indicated values (*p* < 0.0001; Table 2). Furthermore, the second strongest correlation was identified between RBM17 and CHEK2, with the indicated values (*p* < 0.0001).

**Figure 5:**
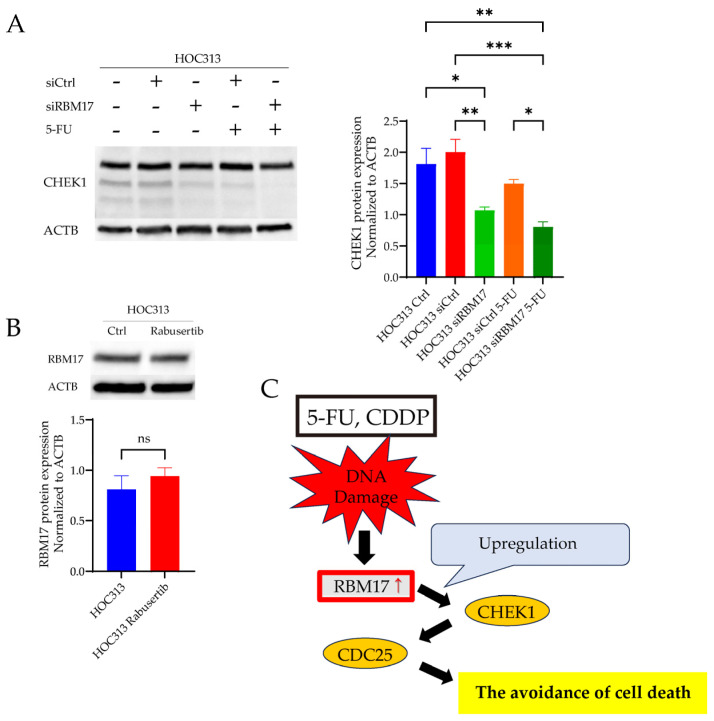
The relationship between RBM17 and CHEK1. (**A**) Upon silencing RBM17 expression using siRNA in the HOC313 cell line, a notable reduction in CHEK1 protein expression was observed through Western blot analysis. A comparison of siCtrl cell lines treated with 5-FU and siRBM17 cell lines treated with 5-FU also revealed that the RBM17 knockdown cell lines demonstrated a significant reduction in CHEK1 protein expression. The experiment had five biological replicates. Quintuplicate blots are shown in the Supplementary Materials (Figure S3A). * *p* < 0.05, ** *p* < 0.01, *** *p* < 0.001. (**B**) The expression of the RBM17 protein in the HOC313 cell line treated with a CHEK1 inhibitor (rabusertib) did not exhibit any alterations. The experiment had three biological replicates. Triplicate blots are shown in the Supplementary Materials (Figure S3B). (**C**) The postulated mechanism of the RBM17-mediated multidrug resistance to cancer chemotherapy. The proposed pathway is based on our two findings: (i) The anticancer drugs 5-FU and CDDP, which cause DNA damage, induce upregulation of the RBM17 gene (up-arrow) and (ii) RBM17 directly or indirectly promotes the expression of a DDR-related gene, CHEK1.

Remarkably, the expression of the CHEK1 protein was reduced by the knockdown of the *RBM17* gene (Figure 5A). When 5-FU was administered to cell lines transfected with RBM17 siRNA (and control siRNA), total CHEK1 expression was significantly decreased in the RBM17 siRNA-treated group compared with the control siRNA-treated group. (Figure 5A, right graph). Furthermore, the addition of a CHEK1 inhibitor did not result in any change in the expression of RBM17 protein (Figure 5B).

## 3. Discussion

RBM17 was reported to play an important role in the development of resistance to multiple anticancer drugs [9,10,11,12]. In this study, we discovered the considerable effects of RBM17 on CHEK1 expression in the ATM/ATR pathway.

We conducted a comparative analysis of the sensitivity of OSCC lines to three anticancer drugs and their levels of RBM17 protein and gene expression. The cell line HOC313, which exhibited high RBM17 mRNA and protein expression, showed low sensitivity to anticancer drugs in our study, indicating that high RBM17 expression, particularly that of mRNA, contributes to the development of resistance to anticancer drugs. Furthermore, cell lines that overexpress the *RBM17* gene exhibit multidrug resistance [14], suggesting that the expression levels of the gene and mRNA (or possibly both gene and protein) of RBM17 must be involved in the multidrug resistance. During the process of measuring the drug sensitivity of the cell lines, we indeed observed that the expressions of the RBM17 gene and protein were elevated in cell lines that had been exposed to anticancer drugs. Because RBM17 functions as a splicing factor and 5-FU and CDDP induce adducts on cellular DNA, we hypothesized that damage to cellular DNA might recruit RBM17 and induce alterations at the transcriptional level via the splicing process. It has been reported that *RBM17* knockdown enhances the sensitivity of hypopharyngeal cancer cells to CDDP [12]. We also demonstrated notable shifts in the sensitivity to the distinct anticancer drug 5-FU in the *RBM17* knockdown cells compared to the control cells.

5-FU and CDDP, but not PTX, were associated with increased RBM17 expression in all three OSCC cell lines tested. Furthermore, siRNA-mediated knockdown of *RBM17* was observed to enhance drug sensitivity to CDDP [12]. Here, we propose that the mechanism by which RBM17 acquires drug resistance would involve the DDR. In DDR-related genes, *CHEK2* stabilizes *TP53* and arrests the cell cycle at the G1/S phase, and the ATR-CHEK1 axis mediates replication fork collapse, protecting cells from replicative stress and mitotic catastrophe that can occur after uncontrolled division [21]. The HOC313 cell line employed in our experiments has a mutation in *TP53* and is characterized by its resistance to chemotherapy [22]. Thus, we assumed that the CHEK2-TP53 axis reaction was unlikely to be implicated and the involvement of the CHEK1-CDC25 axis was plausible. Among the factors involved in the DDR, CHEK1 is considered to play a central role [23]. Moreover, CHEK1 has been reported to play a pivotal role in the ATR-mediated replication stress (RS)-induced checkpoint response, whereby it promotes replication fork stability and the timely repair of DNA double-strand breaks through error-free homologous recombination [18]. Our results of correlation analyses demonstrated a strong positive correlation between RBM17 and CHEK1, suggesting that RBM17 affects DDR-related pathways, particularly through CHEK1. Our research focused on the function of CHEK1 leads us to postulate the potential interplay between RBM17 and CHEK1. We demonstrated that the knockdown of the *RBM17* gene resulted in a reduction in CHEK1 protein expression, while the administration of a CHEK1 inhibitor did not affect the expression of RBM17 protein. Additionally, a significant decrease in total CHEK1 protein expression was observed in RBM17 knockdown cell lines treated with 5-FU compared to 5-FU-untreated controls. These findings suggest that RBM17 promotes the expression of CHEK1 in response to the DDR, either directly or indirectly (Figure 5C). Since RBM17 was identified as a novel splicing factor recently [9,10], we speculate that the expression of *CHEK1* gene or the upstream targeted genes is promoted through enhanced splicing.

Consequently, the prevailing strategy for overcoming resistance entails targeted therapies, which are treatment with the combination of a poly (ADP ribose) polymerase (PARP) inhibitor, other inhibitors of the DDR, and immune-checkpoint inhibition [24]. Among other DDR-related inhibitors, CHEK1 inhibitors are promising candidates, yet the outcomes of clinical trials remain inconclusive. One of the reasons for the lack of progress in the clinical trials is that the DNA damage repair mechanism of ATM or DNA-dependent protein kinase and the cell proliferation effect of *FGFR2* act in a complementary manner to the inhibition of CHEK1 function. Such a complication presents a challenge to the development of effective therapeutic strategies [25]. It is thus evident that further investigation is required into ATM and other factors that lie upstream of the pathway involving CHEK1. Thus, RBM17, which evidently affects CHEK1 expression, could be a promising candidate for therapeutic intervention (Figure 5C).

The use of RBM17 inhibitors may enhance the efficacy of current anticancer drugs during chemotherapy. However, no inhibitors of RBM17 have yet been developed. We assume that drug repositioning to identify drugs capable of inhibiting RBM17 is a viable and effective approach. Furthermore, we hypothesize that the expression of RBM17 in cancerous tissues may serve as a valuable biomarker for predicting the efficacy of existing chemotherapy regimens.

## 4. Materials and Methods

### 4.1. Cell Culture

Five different human OSCC lines, i.e., SAS, OSC19, SCC-4, HSC3-M3, and HOC313, were used in these experiments. SAS, OSC19, and SCC4 were grown in D-MEM/Ham’s F-12 medium (#048-29785, Wako, Osaka, Japan). HSC3-M3 was grown in MEMα medium (#135-15175, Wako). HOC313 was grown in D-MEM medium (#044-29765, Wako, Osaka, Japan). All media contained 10% fetal bovine serum and 1% penicillin–streptomycin solution (#168-23191, Wako, Osaka, Japan). Cells were incubated at 37 °C in a humidified atmosphere with 5% CO_2_.

### 4.2. MTT Assay for Measuring IC50 Values

The sensitivity of various OSCC lines to fluorouracil (5-FU), cisplatin (CDDP), and paclitaxel (PTX) was examined using the half-maximal inhibitory concentration (IC50) values calculated by MTT assay. For each cell line, three 200 µL aliquots of cell suspension with a concentration of 10,000 cells/mL were prepared and transferred to three wells of a 96-well microplate. Background measurements were taken in wells containing only medium. Following a 48 h incubation period, the suspensions were replaced with fresh medium and one of the three anticancer drugs was introduced to each well at concentrations adjusted to 0–300 µM for 5-FU, 0–800 µM for CDDP, and 0–3 µM for PTX. The cells were then cultured for a further 48 h. Subsequently, the media were replaced with 200 µL of fresh medium, followed by the addition of 20 µL of MTT solution and incubation for 4 h. The solution in each well was removed by aspiration and 200 µL of PBS was added. After approximately 1 min, the solution was again removed by aspiration and 200 µL of DMSO was added to each well and completely dissolved by pipetting. The measured absorbance (570 nm) was then quantified using a microplate reader (VARIOSKAN LUX; Thermo Fisher Scientific, Vantaa, Finland).

### 4.3. RNA Extraction and RT-PCR

RNA extraction was conducted using Qiagen’s RNeasy^®^ Mini Kit (Ref. 74104; Lot: 178020974; QIAGEN, Hilden, Germany) for each OSCC line. Reverse transcription to cDNA was then performed with ReverTra Ace™ qPCR RT Master Mix with gDNA Remover (Ref: FSQ-301; Lot: 360600; Toyobo, Osaka, Japan), according to the instructions provided by the manufacturer. For reverse transcription–polymerase chain reaction (RT-PCR) analysis, THUNDERBIRD™ SYBR^®^ qPCR Mix (Ref: QPS-201; Lot: 267000; Toyobo, Osaka, Japan) was selected as the optimal reagent. The comparative quantification of mRNA levels was achieved using the ΔΔCt method, with normalization to ACTB mRNA levels. The forward and reverse primer sequences utilized in this study were as follows: RBM17 forward, 5′-AATTCCTGGTGCCCTGATG-3′; reverse, 5′-ACCCGTCCACCAAAATACCTC-3′; and ACTB forward, 5′-AGAGCTACGAGCTGCCTGAC-3′; reverse, 5′-AGCACTGTGTTGGCGTACAG-3′.

### 4.4. Protein Extraction and Western Blotting Analysis

Proteins from OSCC lines were extracted using RIPA buffer (#R0278, Sigma-Aldrich, Tokyo, Japan) in accordance with the manufacturer’s instructions. The protein concentration was determined by BCA assay using a Pierce™ BCA Protein Assay Kit (Ref: 23227; Lot: UL294774; Thermo Scientific, Rockford, IL, USA), and all samples were adjusted to a concentration of 2 µg/µL. The protein samples were subjected to sodium dodecyl sulfate–polyacrylamide gel electrophoresis (SDS-PAGE), after which the gels were transferred to polyvinylidene difluoride (PVDF) membranes using a Trans-Blot Turbo Transfer System (Bio-Rad, Hercules, CA, USA) in accordance with the manufacturer’s instructions. The membranes were incubated with 5% skim milk powder in Tris-buffered saline with Tween 20 (TBS-T) for 1 h at room temperature. The membranes were then placed in a TBS-T solution containing the respective primary antibodies—anti-RBM17 (1:3000; #13918-1-AP; Lot: 67923; Proteintech Group, Rosemont, IL, USA), anti-CHEK1 (1:3000; #25887-1-AP; Lot: 49854; Proteintech Group, Rosemont, IL, USA), and anti-β-actin (1:3000; #PM053; Lot: 007; MBL, Nagoya, Japan)—with stirring at 4 °C overnight. After washes in TBS-T, the secondary antibody (anti-mouse IgG, HRP-linked whole Ab sheep, #NA931-1ML, Lot: 17925907 or anti-rabbit IgG, HRP-linked whole Ab donkey, #NA934-1ML, Lot: 17271476, 18152529; Cytiva, Marlborough, MA, USA), prepared at a 1:3000 dilution in the same buffer, was added and the mixture was incubated with agitation at room temperature for 60 min. The membranes were rinsed and processed with chemiluminescence reagents, specifically ImmunoStar^®^ Zeta (Ref: 297-72403; Lot: LEN3911, PAH0850; Wako, Osaka, Japan), and imaged using a Fusion Solo S (Vilber Bio Imaging, Paris, France). The experiment was performed with at least three biological replicates. The detected bands were normalized using β-actin as a reference and quantified using the image analysis software ImageJ for comparison [26].

### 4.5. siRNAs and Transfection

Gene knockdown was achieved by siRNA duplex-mediated RNA interference using Lipofectamine™ RNAiMAX Transfection Reagent (Invitrogen/Thermo Fisher Scientific, Carlsbad, CA, USA; Product No. 13778075) and RBM17 Stealth RNAi™ siRNA (siRNA ID: HSS189314; Lot: 65227769; Thermo Fisher Scientific), which are pre-designed and validated siRNA targeting RBM17. As a control, Stealth™ RNAi Negative Control Medium GC Duplex was used (Ref: 452001; Lot: 2697783; Invitrogen/Thermo Fisher Scientific). The transfection was conducted in accordance with the instructions provided by the manufacturer. Following transfection, the cells were cultured for 48 h and used in a series of assays.

### 4.6. Proliferation Assay

The HSC3-M3 and HOC313 cell lines were then transduced with a negative control siRNA and siRNA targeting *RBM17*. Cell numbers were quantified from day 3 to day 7 following seeding. Photographic images were obtained from three randomly selected locations on three dishes for each of control siRNA and *RBM17* siRNA. Cell numbers were calculated by enumerating the cells in each image.

### 4.7. MTT Assay for Chemosensitivity

The HOC313 cell line was exposed to 5-FU at doses ranging from 0 to 521.28 µM, the HSC3-M3 cell line was exposed to 5-FU at doses ranging from 0 to 41.28 µM, and the resulting absorbances were quantified via an MTT assay after a seven-day incubation period. A total of four control and four siRBM17-transfected samples were subjected to analysis in this study, with three replicates. The measured absorbance (570 nm) was quantified using a microplate reader.

### 4.8. Bioinformatics

The correlation analysis was conducted with the use of Data Explorer 1, an online analytical tool accessible via the DepMap portal “https://depmap.org/portal (accessed on 31 July 2024)” [27]. The analysis was processed using the Data Explorer tool with the following settings: Plot Type = Scatter plot, Points = Models, Data Type = Expression, and Feature = Gene for both *X* and *Y* Axis. In order to facilitate the analysis, data from Expression Public 24Q2 were employed.

### 4.9. Statistical Analysis

The data were analyzed using the GraphPad Prism version 10.4.0 software program (GraphPad Software, San Diego, CA, USA). The IC50 values for anticancer drugs in the treatment of oral squamous cell carcinoma were calculated and plotted using the sigmoidal 4PL logistic model. To facilitate a comparison of mRNA expression, the unpaired *t*-test or one-way ANOVA was employed, according to the circumstances of each case. The drug susceptibility testing was conducted with multiple unpaired *t*-tests. A *p*-value of 0.05 or below was considered to indicate statistical significance.

## Figures and Tables

**Table 1:** IC50 for 5-FU, CDDP, and PTX of OSCC cell lines.

Cell Lines	5-FU (µM)	CDDP (µM)	PTX (nM)
SAS	2.29 (1.67–3.23)	26.79 (20.28–35.48)	0.67 (2.53–0.86)
OSC19	2.73 (2.24–3.56)	100.00 (77.45–148.25)	1.51 (1.13–2.03)
SCC4	2.65 (1.40–6.41)	4.73 (3.45–6.49)	0.26 (0.18–0.30)
HSC3-M3	1.29 (0.62–5.09)	2.10 (0.91–5.20)	1.61 (1.50–1.74)
HOC313	16.29 (1.86–142.89)	98.86 (62.09–232.81)	3.36 (2.43–4.59)

5-FU, fluorouracil; CDDP, cisplatin; PTX, paclitaxel; IC50, half maximal (50%) inhibitory concentration. The numbers in parentheses represent a 95% confidence interval.

**Table 2:** The relationship between RBM17 and DDR-related genes.

Gene	Group	Points	Pearson	Spearman	Slope	Intercept	*p*-value
***CHEK1***	All	1517	0.468	0.445	0.601	0.950	<0.001
	OSCC	49	0.402	0.396	0.596	0.133	0.004
	Other	1468	0.451	0.429	0.567	1.20	<0.001
***ATR***	All	1517	0.387	0.367	0.295	2.08	<0.001
	OSCC	49	0.631	0.591	0.475	1.19	<0.001
	Other	1468	0.380	0.360	0.294	2.08	<0.001
***CHEK2***	All	1517	0.328	0.309	0.337	2.14	<0.001
	OSCC	49	0.512	0.496	0.630	0.395	<0.001
	Other	1468	0.314	0.299	0.324	2.22	<0.001
***ATM***	All	1517	0.221	0.182	0.287	2.30	<0.001
	OSCC	49	0.453	0.493	0.566	−0.419	0.001
	Other	1468	0.181	0.150	0.231	2.69	<0.001
***TP53***	All	1517	0.185	0.189	0.328	2.96	<0.001
	OSCC	49	0.105	0.104	0.217	3.16	0.473
	Other	1468	0.177	0.182	0.314	3.06	<0.001
***GADD45A***	All	1517	−0.011	−0.021	−0.019	5.76	0.670
	OSCC	49	0.263	0.244	0.448	3.32	0.068
	Other	1468	−0.015	−0.027	−0.026	5.80	0.572
***MDM2***	All	1517	−0.104	−0.073	−0.188	6.14	<0.001
	OSCC	49	0.208	0.157	0.256	2.85	0.152
	Other	1468	−0.128	−0.092	−0.234	6.46	<0.001
***CDKN1A***	All	1517	−0.232	−0.230	−0.602	9.13	<0.001
	OSCC	49	0.017	−0.055	0.022	5.61	0.907
	Other	1468	−0.234	−0.231	−0.622	9.26	<0.001

## Data Availability

Restrictions apply to the availability of bioinformatics data. Data were obtained from DepMap portal and are available (https://depmap.org/portal) with the permission of DepMap portal. The other raw data supporting the conclusions of this article will be made available by the authors on request.

## Supplementary Materials

The following supporting information can be downloaded at: https://www.mdpi.com/article/10.3390/ijms26073127/s1.
