# Supplementary figures and images for: RBM17 Promotes the Chemoresistance of Oral Squamous Cancer Cells Through Checkpoint Kinase 1
Source: Int J Mol Sci. 2025 Mar 28;26(7):3127. doi: 10.3390/ijms26073127 (PMC11989059; doi:10.3390/ijms26073127)

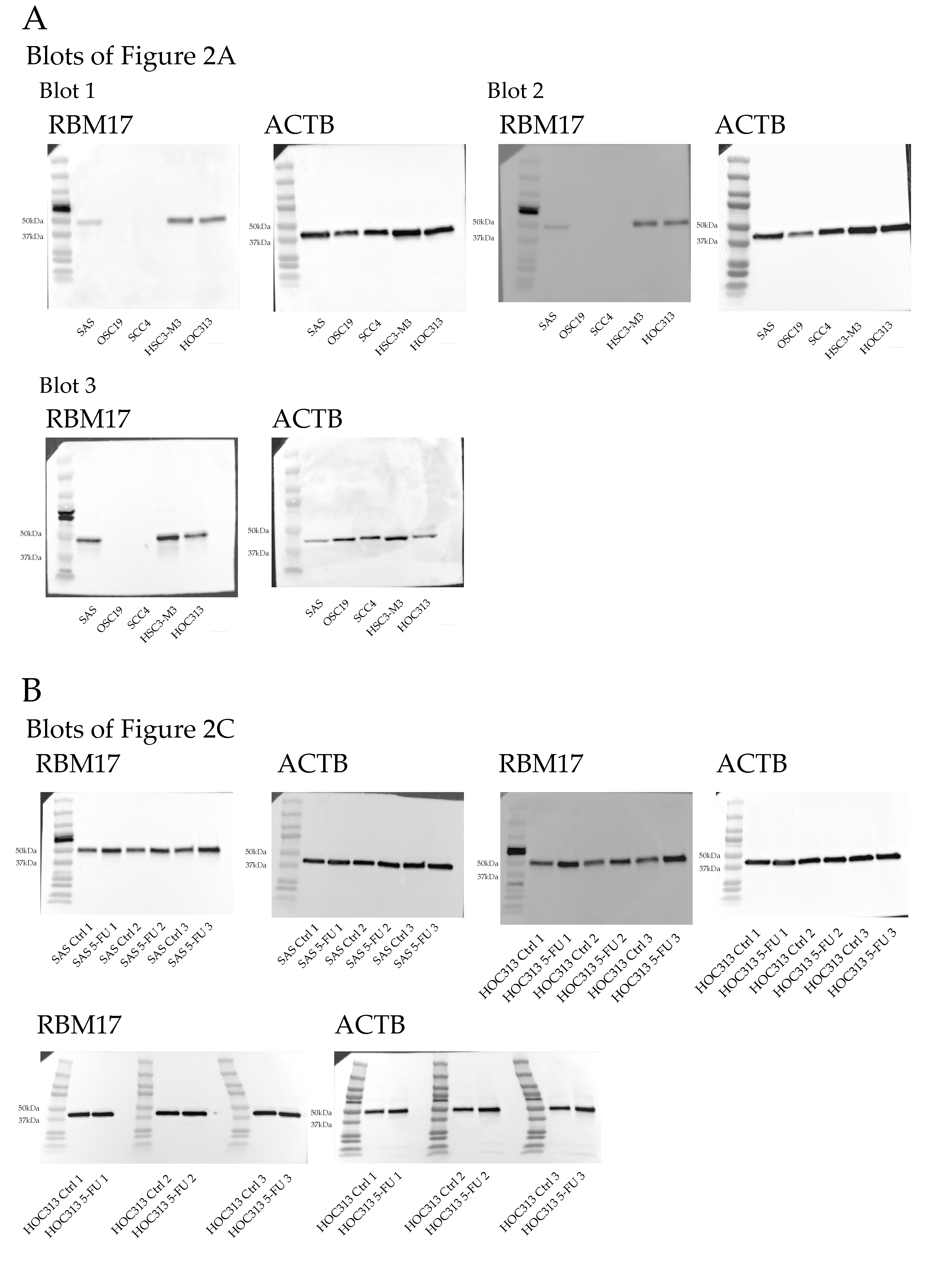

Supplement: Supplementary file 1 [file ijms-26-03127-s001.zip › FigS1.tif]
